# Migration and psychosis: the link between them

**DOI:** 10.1192/j.eurpsy.2022.565

**Published:** 2022-09-01

**Authors:** C. De Andrés Lobo, T. Jiménez Aparicio, C. Vallecillo Adame, A. Gonzaga Ramírez, G. Guerra Valera, I. Santos Carrasco, J. Gonçalves Cerejeira, M. Fernández Lozano, B. Rodríguez Rodríguez, N. Navarro Barriga, M.J. Mateos Sexmero, N. De Uribe Viloria

**Affiliations:** 1 Hospital Clínico Universitario, Psiquiatría, Valladolid, Spain; 2 Hospital Clínico Universitario de Valladolid, Psychiatry, Valladolid, Spain; 3 Clinical Hospital of Valladolid, Psychiatry, Valladolid, Spain; 4 Hospital Universitario Fundación de Alcorcón, Psiquiatría, Valladolid, Spain

**Keywords:** first psychotic episode, migration, risk factors

## Abstract

**Introduction:**

Migrations are a source of stress for patients, which can have repercussions on their Mental Health. We present the case of a native Senegalese patient who presented a first psychotic episode.

**Objectives:**

Presentation of a clinical case of an immigrant patient with a psychotic disorder.

**Methods:**

Bibliographic review on migration and psychosis by searching for articles in Pubmed.

**Results:**

We present the case of a patient of 20 years, a native of Senegal, who has been living in Spain for 3 months in a shelter home. He has no family or relations in Spain, and only speaks Wolof, presenting serious difficulties in communication with healthcare workers. He came to Hospital with his social worker because strange behaviors had been observed. He presented delusional ideation of self-referential and mystical-religious content, related to “the prophet” and “the need to fulfill a mission”. He also presented auditory hallucinations that he identified as of divine origin, and ordered him to perform behaviors such as picking hairs from the ground and various rituals. He acknowledges cannabis and alcohol use in the previous days. Paliperidone treatment was started. Throughout the admission, he begins to show concern for the state of his relatives in Senegal and the need to send them money.

**Conclusions:**

Multiple studies indicate that migrants are at higher risk of psychosis, specially those from countries where the majority of population was black, according to some series. The challenge lies in understanding the mechanisms underlying this increased incidence, taking into account psychosocial factors such as social isolation and trauma.

**Disclosure:**

No significant relationships.

